# Tapeworm Eggs in a 270 Million-Year-Old Shark Coprolite

**DOI:** 10.1371/journal.pone.0055007

**Published:** 2013-01-30

**Authors:** Paula C. Dentzien-Dias, George Poinar, Ana Emilia Q. de Figueiredo, Ana Carolina L. Pacheco, Bruno L. D. Horn, Cesar L. Schultz

**Affiliations:** 1 Laboratório de Paleontologia e Paleoceanografia, Instituto de Oceanografia, Universidade Federal do Rio Grande, Rio Grande, Brazil; 2 Department of Zoology, Oregon State University, Corvallis, Oregon, United States of America; 3 Instituto de Geociências, Universidade Federal do Rio Grande do Sul, Departamento de Paleontologia e Estratigrafia. Porto Alegre, Brazil; 4 Universidade Federal do Piauí, Campus Senador Helvídio Nunes de Barros, Picos, Piauí. Brazil; University of South Alabama, United States of America

## Abstract

Remains of parasites in vertebrates are rare from the Mesozoic and Paleozoic. Once most parasites that live in – or pass through – the gastrointestinal tract of vertebrates, fossil feces (coprolites) or even intestinal contents (enterolites) can eventually preserve their remains. Here we announce the discovery of a spiral shark coprolite from the Paleozoic bearing a cluster of 93 small oval-elliptical smooth-shelled structures, interpreted as eggs of a tapeworm.The eggs were found in a thin section of an elasmobranch coprolite. Most of the eggs are filled by pyrite and some have a special polar swelling (operculum), suggesting they are non-erupted eggs. One of the eggs contains a probable developing larva. The eggs are approximately 145–155 µm in length and 88–100 µm in width and vary little in size within the cluster. The depositional and morphological features of the eggs closely resemble those of cestodes. Not only do the individual eggs have features of extant tapeworms, but their deposition all together in an elongate segment is typical to modern tapeworm eggs deposited in mature segments (proglottids). This is the earliest fossil record of tapeworm parasitism of vertebrates and establishes a timeline for the evolution of cestodes. This discovery shows that the fossil record of vertebrate intestinal parasites is much older than was hitherto known and that the interaction between tapeworms and vertebrates occurred at least since the Middle-Late Permian.

## Introduction

Paleoparasitology is the study of parasites found in archaeological or paleontological material [1], [2]. Parasite remains consists mostly of eggs and larvae of intestinal parasites [3], mainly helminthes, and can provide important diet and disease information regarding their hosts. Helminthes include nematodes (roundworms), trematodes (flukes), cestodes (tapeworms), and acanthocephalans (thorny-headed worms).

Presently, elasmobranchs carry within their spiral intestines various types of parasites, being cestodes the most diverse of them [4]. Cestodes have also been reported from the viscera and body cavity of numerous large teleosts and from the stomach of sharks [5]. Cestodes eggs are characterized by their smooth external surface, mammillations, equatorial bulges, spines or striations [6].

Extant and fossil tapeworm eggs are morphologically very similar to each other and it is impossible to diagnose a specific infection based only on eggs [3] so that paleoparasitological analyses using them are limited to the phylum or ordinal level [7].

Helminth parasites rarely produce eggs with long-lived resistance to environmental stressors. Most of their eggs are fragile, so that they start to decompose very early outside their host [3]. Eggs of some nematode and cestode parasites have a good chance of recovery [3]. The crucial factor for the preservation of parasite eggs is the rapid interruption of decay. It usually occurs only under extreme moist, arid, frozen or anoxic environmental conditions [7].

In archeological studies is usual to find well-preserved remains of intestinal parasites and pathogens which affected health [3], [8], [9], [10]. However, the older the material, the greater the loss of parasites [9]. In fact, the occurrence of fossil parasites in paleontological material is rare. For the Mesozoic [11], just a single record of intestinal parasites (protozoan cysts and helminth eggs) in a coprolite was described, while in the Paleozoic, a mass of possible helminth eggs from the coprolitic rectal fill of a Pennsylvanian shark, perhaps of cestode origin [12], was described. In addition, circlets of parasitic platyhelminth hooks were found in acanthodians from the Late Devonian [13].

Here in we describe the first definite record of cestode parasites in an elasmobranch coprolite from the Paleozoic (about 270 Ma), which is, in fact, the oldest record of parasite eggs in a vertebrate coprolite. The specimen is housed in the Laboratório de Paleontologia de Vertebrados of the Universidade Federal do Rio Grande do Sul, under the collection number UFRGS-PV-429-P. No specific permits were required for the described field studies.

## Materials and Methods

The material came from the Rio do Rasto Formation from Paraná Basin, Mid to Late Permian [14] and was collected in the municipality of São Gabriel, southern Brazil. This formation is characterized by a sequence of fine to medium cross stratificated sandstones, interbedded with siltstones and mudstones, and is interpreted as deposited under fluvio-lacustrine conditions [14]. Its fossil record consists of continental plants, invertebrates and vertebrates [15], [16], [17].

**Figure 1:**
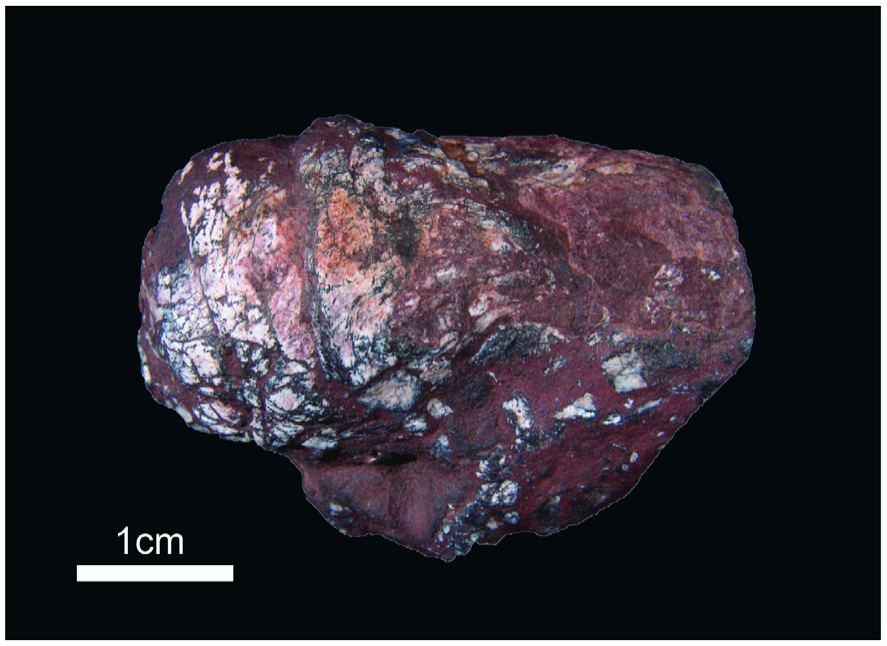
Spiral heteropolar coprolite with cestode eggs.

In the same outcrop where UFRGS-PV-429-P was collected, we have found around 500 other coprolites in an area of 100 m×30 m [18]. All the specimens were photographed and measured and 14 specimens were cut using standard thin section techniques (the same used for rock samples), in order to search for internal structures, petrographic fabrics and inclusions [18], [19]. Longitudinal sections were made in all the 14 selected samples, approximately in a median plane of each one. In three of them a transversal section were made too. The thin section obtained from the longitudinal cutting of UFRGS-PV-429-P showed, under optic microscopy, scales and bones fragments [18], as well as a cluster of unusual oval-shaped structures. This coprolite (Fig. 1), with 5 cm in length and 2 cm in diameter, is classified as a spiral heteropolar [20], characterized by a variable number of closely spaced whorls concentrated in just one end. This morphology and the inclusions (fish scales and bone fragments) are typical features for elasmobranche coprolites [20].

## Results and Discussion

**Figure 2:**
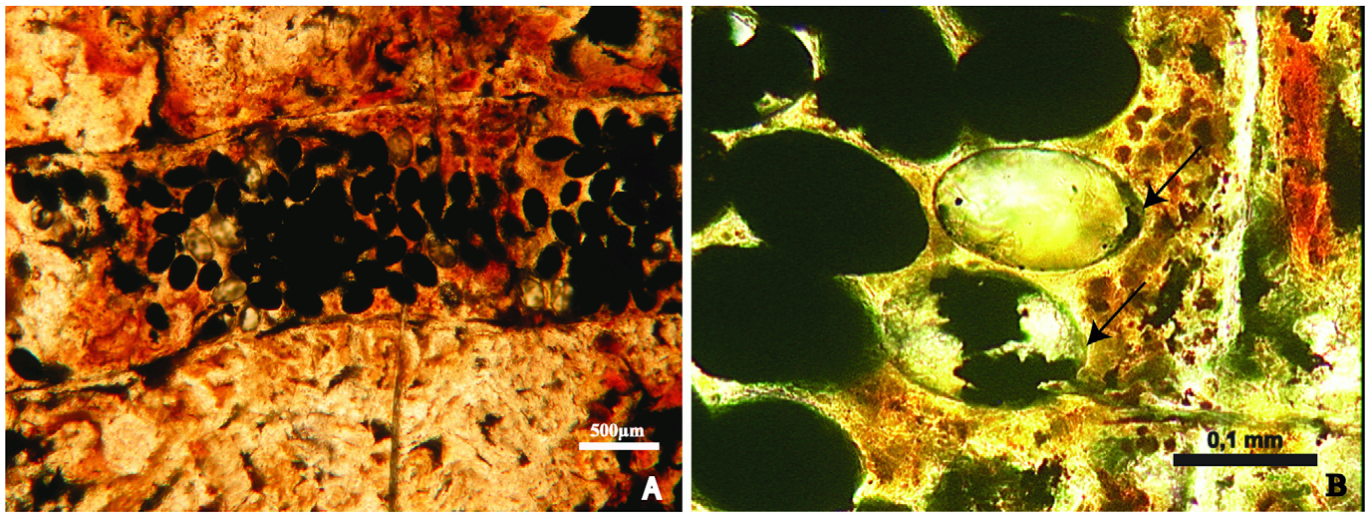
Parasite eggs in a shark coprolite. A - Thin section of the coprolite part containing clustered parasite eggs. B – Cestode eggs, the perfect oval shape hole were formed after the filling were reaped out from the coprolite during the lamination, the arrows show the operculum.

**Figure 3:**
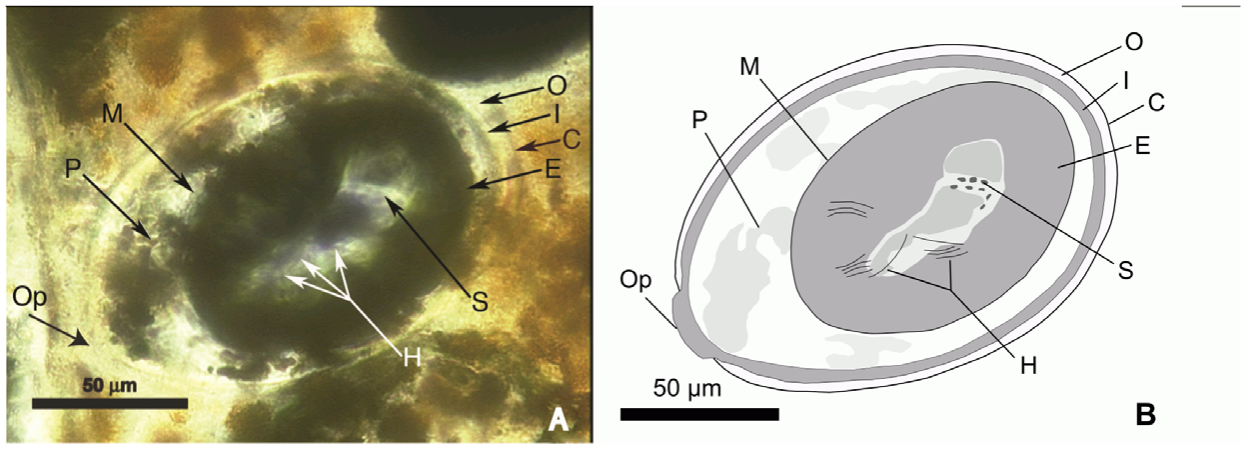
Cestode egg. A - (photo) Cestode egg with a developing embryophore. B - (drawing) Partial reconstruction of egg in A. Abbreviations: C =  capsule or shell; E =  embryophore (ochosphere); H =  putative developing hooklets; I = inner envelope; M = oncospheral membrane; O = outer envelope; P = putative polar thickening; Op = operculum; S = somatic cells.

The oval-shaped structures (#  = 93) are grouped in a segment 4 mm long and 1 mm wide (Fig. 2A). They reveal structures typical of tapeworm eggs. The eggs are ovoid, smooth shelled and range from 145–155 µm in length and 88–100 µm in width. Most are dark and filled with pyrite and/or hematite. Some eggs appear to have been broken (Fig. 2B). One egg contains a developing embryophore (Fig. 3). In this egg, only part of the outer envelope, composed by a thin shell or “capsule”, remains. The remainder, including the vitelline capsule, apparently underwent apoptosis, as occurs with extant cestode eggs [21]. The inner envelope is composed of several layers, the innermost one of which forms the embryophore (oncosphere). Portions of the oncospheral membrane are also apparent. Within the embryophore is a cluster of small putative somatic (or germinative) cells and some fiber-like objects that could represent early stages of hooklet formation. Strands of dense material seemingly attached to the embryophore may be polar thickenings of the inner envelope [22]. A small slightly protruding operculum is also present. Opercula can be better observed on eggs in Fig. 2B.

Besides the similar morphology, the mass deposition of these fossil eggs in an elongate segment is typical of modern tapeworm eggs deposited in mature proglottids. When an extant tapeworm proglottid is full of eggs, it breaks off in the stomach or intestine of the host and eventually passes out of the body with the feces [23]. Normally, maturation of the eggs occurs only after this separation, so fully mature eggs occur only in the host gut and feces [24].

Since the fossil egg contains both yolk and a well-developed shell, there was probably an extensive vitellaria. This is characteristic of the pseudophyllidean egg type, which occurs in the Pseudophyllidea, Trypanorhyncha and Tetraphyllidea, all of which infect aquatic hosts [21], [25], [26].

Tapeworm taxonomy is confusing and controversial. Typically four (out of 11) orders of tapeworms parasitize elasmobranchs, the Diphyllidea, Lecanicephalidea, Tetraphyllidea and Trypanorhyncha [6], [27]. Unfortunately very little information is available on egg structure in these orders. Even the few measurements given are in question since they were made on eggs in segments still attached to the parasite and in many cases, the eggs continue to develop (and enlarge) after the proglottids are released into the gut of the host [24].

The larger size of the fossil eggs described herein distinguish them from known extant tapeworm eggs. In size and shape the fossil eggs most closely resemble those of the shark parasite, *Disculiceps pileatum* (Linton) ( = *Discocephalum pileatum* Linton, 1890), which has oval, brown eggs measuring 110 µm long by 80 µm wide [24]. However the systematic placement of this species is controversial. It was considered a “dubious species” and originally described as a new order, the Heterophyllidea [24], and later was transferred it to the Tetraphyllidea [25]. Since the size of the great majority of elasmobranch tapeworm eggs are unknown, some extant forms could have eggs within the range of the fossils. Then again, the large egg size could be characteristic of some Permian cestodes.

Although it is not possible to assign the fossil eggs to any extant tapeworm group, some characters (operculum, egg shape and size) are reminiscent to those found in the Tetraphyllidea. This is the most widespread order of cestodes found in Elasmobranchs, with some 540 extant species.

In some fish parasites (nematodes species) the mature eggs contain fully formed first-stage larvae, which do not hatch spontaneously in the external environment. Some eggs of other groups are uncleaved at the time of oviposition, and the larvae develop only in the external environment, where they undergo their first moult inside the egg shells and hatch in the external environment [28]. This could explain why we do not observe the larvae in all of the eggs.

The presence of pyrite in the coprolites indicates anoxic environmental conditions, which probably were responsible for the preservation of both coprolites and parasites. The same pattern occurs in feces of Neolithic Age, in which some parasite eggs show the embryo inside the egg, filled with crystals of pyrite [9], [29].

The possibility of the fossil eggs belonging to a trematode or nematode was considered. However, eggs of digenetic trematodes are deposited singly and not in groups as in the case of the present fossils. They also do not demonstrate hook formation during development. Most trematodes associated with sharks are ectoparasitic or occur in the body cavity (pericardial and coelomic cavities). While the spiral valve of extant sharks is usually full of cestodes, there are almost never digenean trematodes in this location [30], [31], [32].

Nematodes are also rare in sharks and while species of Acanthocheilus Molin, 1858 and Ichthyostrongylus Mawson, 1954 occur in the alimentary tract, the eggs are deposited singly, not in masses [33].

The crowding of individuals of a population in a restricted area favors the dissemination of parasites [9], which enhance the possibility of its preservation, as seems to have occurred in the present case. As mentioned above, the coprolite is part of a set of more than 500 specimens found in a restricted area. It was interpreted as a freshwater pond where many fishes became entrapped for some time, probably during a dry period [18]. It could explain the great number of coprolites of different shapes and sizes all together, as well as the anoxia in the bottom of the water column (evidenced by the presence of pyrite in the coprolites and also by the high number of preserved coprolites).

Many extant tapeworms require several hosts to complete their development. The first is often an invertebrate that is eaten by a second host, normally a frog, fish, or reptile, and maturation continues through the next stage [34]. Tapeworms reach adulthood (developing eggs) when that animal is eaten by a third, and final, host. The studied coprolite has fish scales and bone fragments allowing us to infer that the second host of this tapeworm was a fish.

Even in Holocenic remains only a small amount of coprolites contain parasite eggs [8], depend on the method of processing and observation selected. This emphasizes that the finding of parasite eggs in a coprolite of about 270 Ma is an amazing discovery, mainly because no special method was used to find the parasite eggs.

Infectious diseases have been poorly reported for vertebrates from Permian [35] and the Paleozoic as a whole. Even extensive studies of inclusions in coprolites [36] did not reveal any parasite. The fossil parasite eggs presented here corroborate the theory that parasitism was present since the advent of life [37].

This is the earliest fossil record of tapeworm parasitism of vertebrates and establishes a timeline for the evolution of cestodes. Analyses of tapeworm phylogeny state that based on parasite phylogeny and the mapping of host groups, there is no indication that eucestodes existed in archaic sharks and rays [26]. While it is impossible to state what vertebrate group served as the original hosts to tapeworms, the present study shows that elasmobranchs (neoselachians), were hosts of tapeworms some 270 million years ago. The lacustrine environment could well have been the ancestral habitat of cestodes, with elasmobranchs as their primitive final hosts.
